# ReadDB Provides Efficient Storage for Mapped Short Reads

**DOI:** 10.1186/1471-2105-12-278

**Published:** 2011-07-07

**Authors:** P Alexander Rolfe, David K Gifford

**Affiliations:** 1 Computer Science and Artificial Intelligence Laboratory, Massachusetts Institute of Technology, 77 Massachusetts Avenue, Cambridge, MA 02139, USA; 2 Pathogenica, Inc, 18th Floor, 245 First Street, Cambridge, MA 02142, USA

## Abstract

**Background:**

The advent of high-throughput sequencing has enabled sequencing based measurements of cellular function, with an individual measurement potentially consisting of more than 10^8 ^reads. While tools are available for aligning sets of reads to genomes and interpreting the results, fewer tools have been developed to address the storage and retrieval requirements of large collections of aligned datasets. We present ReadDB, a network accessible column store database system for aligned high-throughput read datasets.

**Results:**

ReadDB stores collections of aligned read positions and provides a client interface to support visualization and analysis. ReadDB is implemented as a network server that responds to queries on genomic intervals in an experiment with either the set of contained reads or a histogram based interval summary. Tests on datasets ranging from 10^5 ^to 10^8 ^reads demonstrate that ReadDB performance is generally within a factor of two of local-storage based methods and often three to five times better than other network-based methods.

**Conclusions:**

ReadDB is a high-performance foundation for ChIP-Seq and RNA-Seq analysis. The client-server model provides convenient access to compute cluster nodes or desktop visualization software without requiring a shared network filesystem or large amounts of local storage. The client code provides a simple interface for fast data access to visualization or analysis. ReadDB provides a new way to store genome-aligned reads for use in applications where read sequence and alignment mismatches are not needed.

## Background

Next generation DNA sequencing technology [1] has enabled the use of sequencing to query biological function using methods such as ChIP Seq [2] and RNA Seq [3]. The cost of sequencing is rapidly declining, and as a consequence large repositories of sequencing data have arisen from key biological experiments. Early experiments used 25 bp tags for ChIP-Seq and generated a few million reads per sample. Recently available long reads, paired reads, and read counts over 10^8 ^reads per sample have enabled RNA-Seq [3,4] experiments that detect splice form variants. Deep sequencing also permits detection of SNPs and other variants across populations and between samples and reference sequences.

The number of public, potentially useful datasets is vast. For example, the ENCODE project (http://www.genome.gov/10005107, [5]) has produced hundreds sequencing datasets in human cell lines for histone modifications and transcription factor binding. Analyses incorporating these datasets might query the genome in kilobase windows and perform millions of queries against each dataset in an analysis. The full alignment output for a dataset might be hundreds of megabytes to several gigabytes (see Table 1), requiring over a terabyte of space for the complete ENCODE datasets.

**Table 1:** Storage space required for each format

	small	medium	large
read set size	536 k	13.9 m	175 m
BigWig	989 kB	23 MB	83 MB
ReadDB	3.3 MB	126 MB	463 MB
BAM	25 MB	532 MB	5.1 GB

Space used by ReadDB, BigWig files, and BAM files (including the index files for each format).

The data requirements for analysis algorithms vary by application. Assembly and SNP detection algorithms require access to all bases of the read and the quality score of each base. In contrast, some ChIP-Seq analysis algorithms can ignore individual reads and operate only on the histogram of read depth at each base. Common formats currently include BAM [6] and WIG/BigWig [7]. BAM stores the full alignment output, including the read sequence, read quality, hit position, and mismatch and indel information. WIG (and its binary version BigWig) is a histogram format that stores positions and values; each position corresponds to a single histogram bin and the value to the number of reads starting in or crossing that bin.

Our software, ReadDB, aims to support ChIP-Seq, RNA-Seq, DNA methylation, and DNAse hypersensitivity analysis and visualization applications by providing efficient access to mapped read positions for single and paired end reads. ReadDB operates as a network server process such that data can be curated by one or a few people for a large group and so that clients do not need access to a shared network filesystem or large amounts of local disk space. The server can respond to queries either with the positions of individual reads or with a histogram generated with arbitrary bin size. The provided client code can then provide quick network access to thousands of datasets. However, ReadDB does not implement analysis algorithms (eg, ChIP-Seq peak calling or RNA-Seq transcript detection) or a visualizer itself. Rather, we use and expect others to use ReadDB as the backend storage for these applications.

## Implementation

### Architecture

ReadDB implements an abstraction called an *alignment *that describes where reads from a read set map to a reference genome. A *read set *may be a collection of reads from a single experiment, or it may combine reads from multiple experiments that are replicates. Queries on an alignment are performed with respect to the coordinate space of the reference genome used for the alignment. Note that a read set can be aligned to different genomes, or different alignment methods or parameters can be used to align a read set to the same genome; each alignment variant for a given read set is stored as a unique alignment.

**Figure 1:**
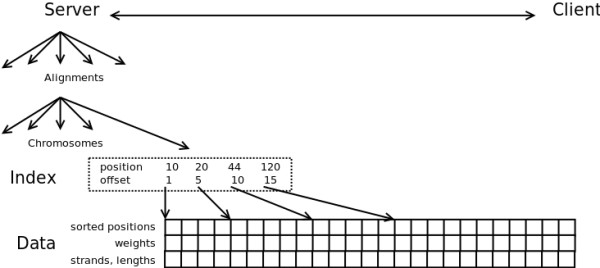
**ReadDB architecture**. ReadDB uses a client-server architecture to give clients access to a large set of mapped read positions. The server stores each set of aligned reads in one directory and uses several files per chromosome. The index files allow quick access to the read data (which is sorted by position) and are small enough that the server can cache many index files in memory at once.

An alignment has distinct data structures for each chromosome. Three files make up the column store and hold the hits (a hit is a genomic position to which a read is mapped) sorted by position (5' coordinate of the aligned read, a 32 bit int), the strand and hit length (one bit strand, 15 bit hit length), and weight (32 bit float, typically the inverse of the number of alignments for the read). A fourth file indexes the sorted hits. Figure 1 provides an overview of the ReadDB architecture.

ReadDB's key feature is the index of the sorted hits for a chromosome. The index file is similar to a single block of a B-tree index and points into the main data files every 4-16 kb (disk reads smaller than this won't be significantly faster so a denser index doesn't yield a performance improvement). At four bytes per record in the data file and eight bytes per entry in the index, a 64 kb index file is sufficient for eight million ((4 kb/4) * (64 kb/8)) to thirty two million records. As most experiments contain fewer than ten million hits per chromosome, most index files are smaller than 64 kb and ReadDB can cache hundreds of index files in a reasonable amount of memory. The index provides *O*(*log*(*n*)) accesses into the list of hits, meaning that ReadDB query operations are *O*(*log*(*n*) + *m*) where *n *is the number of hits stored and *m *is the number of hits returned.

For compactness, ReadDB stores chromosomes as integers. Client code may either use this directly to store chromosome numbers (with some alternate scheme to handle X, Y, mt, 5_random, etc) or may use an external database that maps chromosomes to numeric identifiers. In the former case, the alignment name ought to indicate the genome assembly, eg "GM12878 CTCF against mm8." In the latter case, a system such as GSE [8] maps the combined genome build and chromosome name to an identifier and maintains metadata about each alignment. In either case, client code can unambiguously specify which genome should be used and could even query both genomes by executing queries against both chromosome identifiers.

While ReadDB can return individual read positions, many applications benefit from server generated histograms. Condensing a potentially large number of hits into a much smaller number of bin positions and counts generates substantially less network traffic between the client and server. The server can also answer aggregate queries such as the total number of reads or the sum of hit weights in a region, chromosome, or dataset.

ReadDB also supports paired-end read sets and allows queries based on either end of the read. The query specifies, for example, a genomic range for which to query the "left" reads and all read pairs for which the left read maps to that range are returned. Paired-end storage is implemented by adding columns for the mate's position, strand, and length and by storing each read twice: once keyed by the "left" read and once keyed by the "right" read. The duplication allows for quick access by either side at a minimal cost of storage space since the data stored per read is only slightly larger than the corresponding key (chromosome, position) and pointer.

### Interface

The ReadDB server accepts queries as text and may be queried from any programming language. The interface, described in full in additional file 1, includes methods to

• store single-end and paired-end hits to an alignment. Both hit types may be stored to an alignment

• delete hits from an alignment (single or paired-end)

• retrieve a list of chromosomes to which reads were mapped in an alignment

• retrieve the number of hits present in an alignment or in a particular chromosomal region of an alignment

• retrieve the hits present in an alignment or in a particular chromosomal region of an alignment. The interface allows only positions or weighs to be retrieved in addition to the full hit information.

• retrieve a histogram either of hit counts or hit weight sums across a chromosomal region. The interface allows the caller to specify the histogram bin width.

All queries may be filtered by hit strand, paired-endness, and weight.

## Results

We tested a Java ReadDB client with a remote ReadDB server against the following alternatives:

• Remote BAM (HTTP access) and remote BAM index (HTTP access)

• BAM and index on local disk

• Remote BigWig (HTTP access)

• BigWig on local disk

ReadDB and BAM files were queried to retrieve individual read positions. BigWig files only support queries for histograms and were tested in this mode.

For each setup, we tested results from aligning three read files to the hg19 assembly: a small set of 513,870 Pol2 ChIP-Seq reads [9], a medium set of 13,901,600 CTCF ChIP-Seq reads (ENCODE project, Crawford Lab at Duke University, ftp://hgdownload.cse.ucsc.edu/goldenPath/hg18/encodeDCC/wgEncodeChromatinMap/wgEncodeUtaChIPseqRawDataRep3K562Ctcf.fastq.gz), and a large set of 175,128,655 reads DNAse hypersensitivity reads (ENCODE project, Stam/Uw Lab at the University of Washington, http://hgdownload.cse.ucsc.edu/goldenPath/hg18/encodeDCC/wgEncodeUwDnaseSeq/wgEncodeUwDnaseSeqRawDataRep2Gm12878.fastq.gz). Table 1 shows the number of reads in each dataset as well as the storage space required for each method.

Each test consisted of retrieving all hit positions within some number of regions *n *(ranging from *n *= 10 to *n *= 100000) of size 1 kb, 10 kb, or 100 kb. ReadDB was queried with our Java code. BAM files were queried with code using the Picard http://picard.sourceforge.net library and BigWig files were queried with Perl code using the Bio::BigFile module (http://search.cpan.org/~lds/Bio-BigFile-1.06; we were unable to find a Java interface for the BigWig file format). Our ReadDB server machine also provided HTTP access to the BAM and BigWig files off the same filesystem that provides ReadDB storage using Apache 2.2.12.

**Figure 2:**
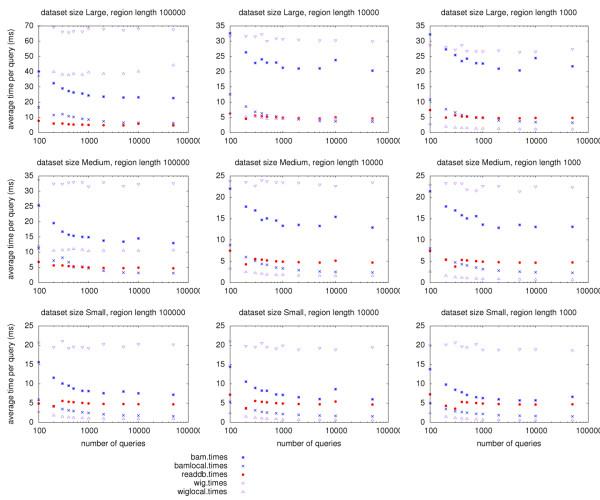
**ReadDB performance compared to other formats**. ReadDB's performance nearly matches that of local BAM or BigWig files on the medium and large datasets. Each method was used to query *n *randomly chosen regions (of size 1 kb, 10 kb, or 100 kb) and the resulting average query time is shown in milliseconds. The client and server machines were connected by gigabit ethernet. ReadDB and the BAM tests retrieved individual hits; BigWig retrieved a histogram in 10 bp bins as the BigWig format cannot store individual read positions. bamlocal and wiglocal queried files on local disks rather than on a remote server.

Over a local area gigabit network, remote ReadDB performs similarly to local-disk access and three to five times faster than remote BAM or BigWig files. As shown in Figure 2, ReadDB also outperforms remote BAM and remote WIG files for all dataset sizes and query region sizes. ReadDB also outperforms a local BAM file in some tests against the large dataset. As expected, the local WIG file often provides good performance as one would expect from a local data source that is a pre-computed histogram.

**Figure 3:**
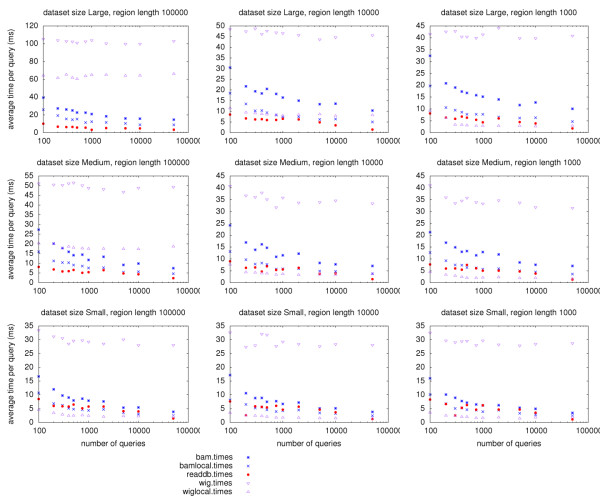
**Performance comparison with no network latency**. Comparison of runtimes with the client software and server on the same machine. To separate the effects of network throughput and latency from those of the disk and CPU, we ran the test suite from the server machine. The ReadDB test still went through the ReadDB server and the BAM and WIG files were read by the httpd process. These tests indicate that ReadDB's fast queries compared to remote BAM are not the result of BAM queries passing more information across the network; the ReadDB queries through the ReadDB server are faster than either BAM through httpd or BAM read directly from local disk.

Tests performed through the network server (ReadDB or HTTP) from the server machine itself help separate the effect of file format from the effects of network throughput and latency. As shown in Figure 3, ReadDB access in this situation is as at least as fast as local BAM access indicating that ReadDB's on-disk format provides a performance advantage over the BAM format as the dataset size increases. The difference between local BAM performance and BAM via HTTP in this test indicates the extra overhead incurred by the HTTP server and avoided by ReadDB's server.

**Figure 4:**
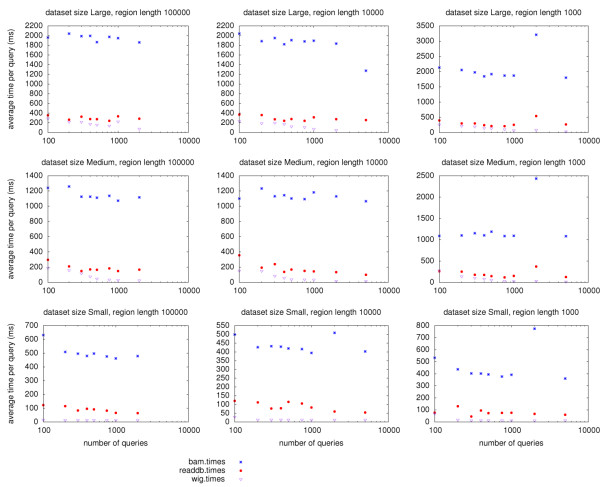
**Performance comparison across a high-latency network**. Comparison of runtimes across a residential broadband connection. The query times correlate with the amount of data transfered over this slower connection-the histogrammed BigWig results are fastest, followed by the per-read ReadDB results (readdb) followed by the BAM results. The server-side histograms with ReadDB (readdb.histogram) demonstrate the best performance as they combine histogrammed data transmission with the fast ReadDB server. Each method was used to query *n *randomly chosen 1 kb regions; only a single replicate was run. The readdb. histogram test used server-generated histograms such that the data passed across the network is similar to the BigWig format rather than the per-read format in the bamlocal and readdb tests.

We also performed a subset of tests from a residential broadband connection (10 Mb/s down, 1 Mb/s up, roughly 20 ms latency to the server) to determine the effects of higher latency and lower bandwidth. As seen in Figure 4, runtimes increased for all methods. The difference between ReadDB and BAM increased substantially to five to seven fold. WIG performed very well in this test as its relatively small size incurred the least network transmission time.

## Conclusions

ReadDB's query performance bests that of remote BAM and BigWig files on all but the smallest datasets. Since ReadDB's theoretical query time is *O*(*log*(*n*) + *m*) (where *n *is the number of hits stored and *m *is the number of hits returned), ReadDB should scale to datasets that are many times larger than those evaluated here. Our tests demonstrate that the expected behavior holds over two orders of magnitude in dataset size.

ReadDB provides fast and compact access to aligned short-read datasets in situations where mismatch information and quality scores are unnecessary. In particular, we have found that ReadDB provides an excellent back end for visualization and analysis of ChIP-Seq, DNA methylation, DNAse hypersensitivity, and RNA-Seq datasets. We currently store over four thousand alignments covering over two thousand lanes of sequencing and enjoy performance nearing that of local disk without incurring the local storage overhead of BAM or BigWig files.

## Competing interests

The authors declare that they have no competing interests.

## Availability and Requirements

readdb.jar, provided as additional file 2, contains the Java class files, source files, and a HOWTO file describing how to setup the ReadDB server. ReadDB requires Java 1.6 to run. The source code is provided under the GPL version 3. Updated versions of the jar file are available at http://cgs.csail.mit.edu/readdb.

## Authors' contributions

AR designed and wrote the software and performed the comparisons to other storage methods. DKG provided design suggestions and helped designed the comparison methods. AR and DKG wrote the paper and read and approved the final version.

## Supplementary Material

Additional file 1 **A description of the ReadDB interface and the method calls it contains**. Click here for file

Additional file 2 **The Java class files, source files, and a HOWTO file describing how to setup the ReadDB server**. Updated versions of the jar file are available at http://cgs.csail.mit.edu/readdb. Click here for file
