# Supplementary material for: ReadDB Provides Efficient Storage for Mapped Short Reads
Source: BMC Bioinformatics. 2011 Jul 7;12:278. doi: 10.1186/1471-2105-12-278 (PMC3143109; doi:10.1186/1471-2105-12-278)
Supplement: Additional file 1 — A description of the ReadDB interface and the method calls it contains. [file 1471-2105-12-278-S1.PDF]

ReadDB Interface

Here we present the interface as a set of Java method signatures, though ReadDB accepts queries as text and may be queried from any language. Java's object types Boolean and Integer are used for optional parameters- if the value is null, then the query does not filter on that field. Common parameters include

- `paired` indicates whether to query paired-end or single-end hits
- `isLeft` indicates whether to query based on the left read or right read of a paired-end read
- `plusStrand` indicates that only hits on the plus strand should be returned
- `startpos` indicates the beginning of a genomic interval from which hits should be returned
- `stoppos` indicates the end of of a genomic interval from which hits should be returned
- `minimumHitWeight` filters hits based on their weight (typically the inverse of the number of genomic matches)

ReadDB uses two special data classes. `SingleHit` represents a single-ended read mapped to the genome and contains a chromosome, position, strand, length, and weight. `PairedHit` represents a pair of reads mapped to the genome and contains a left chromosome, left position, left strand, left length, right chromosome, right position, right strand, right length, and combined weight.

The ReadDB client code provides these method calls which correspond to the ReadDB server interface.

- `new(String serverhostname, int serverport, String username, String password)` creates a new connection
- `void storeSingle(String alignmentname, List<SingleHit>)` adds the list of unpaired hits to the set of hits stored for the alignment
- `void storePaired(String alignmentname, List<PairedHit>)` add the list of paired-end hits to the set of hits stored for the alignment
- `boolean exists(String alignmentname)` determines whether the alignment exists in ReadDB

- void deleteAlignment(String alignmentname, Boolean paired) deletes hits from the alignment

- Set<Integer> getChroms(String aligmentname, boolean paired, Boolean isLeft) returns the set of chromosomes that exist in this alignment

- int getCount(String alignmentname, boolean paired, Boolean isLeft, Boolean plusStrand) returns the total number of hits in this alignment

- float getWeight(String alignmentname, boolean paired, Boolean isLeft, Boolean plusStrand) returns the sum of the hit weights in this alignment

- int getCount(String alignmentname, int chromosome, boolean paired, Integer startpos,Integer stoppos, Float minimumHitWeight, Boolean isLeft, Boolean plusStrand) returns the number of hits that meet the query criteria

- float getWeight(String alignmentname, int chromosome, boolean paired, Integer startpos,Integer stoppos, Float minimumHitWeight, Boolean isLeft, Boolean plusStrand) returns the sum of the weights of the hits that meet the query criteria

- int[] getPositions(String alignmentname, int chromosome, boolean paired, Integer startpos, Integer stoppos, Float minimumHitWeight, Boolean isLeft, Boolean plusStrand) returns the positions of the hits that meet the query criteria

- float[] getWeights(String alignmentname, int chromosome, boolean paired, Integer startpos, Integer stoppos, Float minimumHitWeight, Boolean isLeft, Boolean plusStrand) returns the weights of the hits that meet the query criteria. getPositions() and getWeights() return values in the same order, ie, the values could be paired to produce the positions and weights of the hits that were stored.

- List<SingleHit> getSingleHits(String alignmentname, int chromosome, boolean isLeft, Integer startpos, Integer stoppos, Float minimumHitWeight, Boolean plusStrand) returns the set of SingleHit objects meeting the query criteria

- List<PairedHit> getPairedHits(String alignmentname, int chromosome, boolean isLeft, Integer startpos, Integer stoppos, Float minimumHitWeight, Boolean plusStrand) returns the set of PairedHit objects meeting the query criteria

- Map<Integer,Integer> getHistogram(String alignmentname, int chromosome, boolean paired, boolean extendHitsByTheirLength, int binsize, Integer startpos, Integer stoppos, Float minimumHitWeight, Boolean plusStrand) returns a histogram computed over alignment positions. extendReadsByTheirLength indicates whether a hit should contribute to a single bin (false) or whether it should contribute to each bin between the hit's position and the position plus the hit length.

- `Map<Integer,Float> getWeightHistogram(String alignmentname, int chromosome, boolean paired, boolean extendHitsByTheirLength, int binsize, Integer startpos, Integer stoppos, Float minimumHitWeight, Boolean plusStrand)` returns a histogram computed over hit weights positions.

- `shutdown()` requests the server to shut down

- `Map<String, Set<String>> getACL(String alignmentname)` returns the ACL for an alignment. The ACL is three lists: the set of users and groups who can read the alignment, the set who can write to the alignment, and the set who can change the ACL for the alignment.

- `void setACL(String alignmentname, Set<ACLChangeEntry>` makes a set of changes to the alignments ACL.

- `void addToGroup(String username, String group)` adds a user to a group.
